# Supplementary material for: Reconciling Mining with the Conservation of Cave Biodiversity: A Quantitative Baseline to Help Establish Conservation Priorities
Source: PLoS One. 2016 Dec 20;11(12):e0168348. doi: 10.1371/journal.pone.0168348 (PMC5173368; doi:10.1371/journal.pone.0168348)
Supplement: S1 Dataset — (ZIP) [file pone.0168348.s002.zip › Taxa/Serra Norte/SN_2013/N4WS/Lista N4WS-19.pdf]

CAVIDADE N4WS-0019

Classe	Ordem	Fam/Outros	Gên/Outros	Espécie	S-U	U-S	Zon.
Arachnida	Acari	(Oribatida)		sp.2		X	E
Arachnida	Acari	(Parasitengonina)		sp.3		X	E
Arachnida	Acari	Argasidae	*Ornithodoros*	sp.1	X		P
Arachnida	Acari	Ixodidae	*Amblyomma*	sp.1	X	X	E/P
Arachnida	Acari	Tarsonemidae		sp.1	X		P
Arachnida	Amblypygi	Phrynidae	*Heterophrynus*	*longicornis*	3	1	P
Arachnida	Araneae	Araneidae		jovem	X	X	E
Arachnida	Araneae	Filistatidae		sp.1		X	E
Arachnida	Araneae	Mysmenidae	*Microdipoena*	sp.1	X		E
Arachnida	Araneae	Ochyroceratidae		jovem		X	E
Arachnida	Araneae	Oonopidae	aff. *Triaeris*	sp.1	X	X	P
Arachnida	Araneae	Pholcidae	*Mesabolivar*	*eberhard*	X	X	E
Arachnida	Araneae	Prodidomidae	*Lygromma*	sp.2	X		E
Arachnida	Araneae	Salticidae	*Freya*	sp.1	X		E
Arachnida	Araneae	Scytodidae	*Scytodes*	*globula*		1	E
Arachnida	Opiliones	Cosmetidae	*Roquettea*	*singularis*	2		E
Arachnida	Opiliones	Sclerosomatidae	*Prionostema*	sp.1	X	X	E
Arachnida	Pseudoscorpiones	Chernetidae	*Spelaeochernes*	sp.1	X	X	E/P
Arachnida	Pseudoscorpiones	Chthoniidae		jovem	X		P
Arachnida	Ricinulei	Ricinoididae	*Cryptocellus*	*tarsilae*		X	E
Arachnida	Schizomida	Hubbardiidae	aff. *Rowlandius*	sp.1		X	E
Diplopoda	Polydesmida	Chelodesmidae		sp.1		1	E
Entognatha	Collembola	Paronellidae		sp.1		X	E
Entognatha	Diplura	Campodeidae		sp.1		X	E
Insecta	Blattodea	Blaberidae		jovem		3	E
Insecta	Coleoptera	Staphylinidae	Pselaphinae	sp.38		X	E
Insecta	Diptera	Ceratopogonidae		sp.	X	X	E
Insecta	Diptera	Chloropidae		sp.	X		E
Insecta	Diptera	Culicidae	*Culex*	sp.	X		E
Insecta	Diptera	Muscidae		sp.	X		P
Insecta	Diptera	Mycetophilidae	*Aphrastomyia*	sp.	X		E
Insecta	Diptera	Psychodidae	*Micropygomyia*	*pilosa*		X	E
Insecta	Diptera	Psychodidae	*Pintomyia*	*gruta*	X		E
Insecta	Diptera	Psychodidae	*Sciopemyia*	*sordellii*		X	E
Insecta	Diptera	Stratiomyidae		sp.	X		P
Insecta	Diptera	Tachinidae	Dexiinae	sp.	X		E
Insecta	Diptera	Tipulidae	Tipulinae	sp.	X		E
Insecta	Heteroptera	Miridae		jovem	X	X	E
Insecta	Heteroptera	Reduviidae	Emesinae	jovem		2	E
Insecta	Heteroptera	Reduviidae	Reduviinae	jovem	1	2	E
Insecta	Homoptera	Cixiidae		sp.3	X	X	E
Insecta	Hymenoptera	Braconidae		sp.1	X		E
Insecta	Hymenoptera	Formicidae	*Dolichoderus*	aff. *bispinosus*	X		E/P
Insecta	Hymenoptera	Formicidae	*Hypoponera*	sp.1		X	E
Insecta	Isoptera	Termitidae	*Nasutitermes*	sp.	X		E/P
Insecta	Lepidoptera	Gelechioidea		sp.3		X	E
Insecta	Lepidoptera	Noctuoidea		sp.1	1		E
Insecta	Lepidoptera	Noctuoidea		sp.2	X		E
Insecta	Lepidoptera	Noctuoidea		sp.9	X		E

Insecta	Orthoptera	Phalangopsidae	*Paraclodes*	sp.	17	1	E
Insecta	Orthoptera	Phalangopsidae	*Phalangopsis*	sp.	12	30	P
Insecta	Orthoptera	Phalangopsidae		sp.2		1	E
Insecta	Psocoptera	Epipsocidae	*Epipsocus*	sp.2		X	E
Pauropoda	Tetramerocerata			sp.1		X	E
Symphyla		Scutigerellidae	*Hanseniella*	sp.1	X		P
Amphibia	Anura	Strabomantidae	*Pristimantis*	cf. *fenestratus*	9		E/P
Mammalia	Chiroptera	Emballonuridae	*Peropteryx*	sp.	2		E/P
